# The use of SWATH to analyse the dynamic changes of bacterial proteome of carbapanemase-producing *Escherichia coli* under antibiotic pressure

**DOI:** 10.1038/s41598-018-21984-9

**Published:** 2018-03-01

**Authors:** Hanna E. Sidjabat, Jolene Gien, David Kvaskoff, Keith Ashman, Kanchan Vaswani, Sarah Reed, Ross P. McGeary, David L. Paterson, Amanda Bordin, Gerhard Schenk

**Affiliations:** 1 Centre for Clinical Research, Faculty of Medicine, The University of Queensland, Royal Brisbane and Women’s Hospital Complex, Herston, Queensland, 4029 Australia; 2 Sciex, 2 Gilda Court, Mulgrave, 3170, Victoria Australia; 3 The University of Queensland, School of Chemistry and Molecular Biosciences, Brisbane, Queensland, 4072 Australia

## Abstract

Antibiotic resistance associated with the clinically significant carbapenemases KPC, NDM and OXA-48 in Enterobacteriaceae is emerging as worldwide. In Australia, IMP-producing Enterobacteriaceae are the most prevalent carbapenemase-producing Enterobacteriaceae (CPE). Genomic characteristics of such CPE are well described, but the corresponding proteome is poorly characterised. We have thus developed a method to analyse dynamic changes in the proteome of CPE under antibiotic pressure. Specifically, we have investigated the effect of meropenem at sub-lethal concentrations to develop a better understanding of how antibiotic pressure leads to resistance. *Escherichia coli* strains producing either NDM-, IMP- or KPC-type carbapenemases were included in this study, and their proteomes were analysed in growth conditions with or without meropenem. The most significant difference in the bacterial proteomes upon the addition of meropenem was triggered amongst NDM-producers and to a lower extent amongst KPC-producers. In particular, HU DNA-binding proteins, the GroEL/GroES chaperonin complex and GrpE proteins were overexpressed. These proteins may thus contribute to the better adaptability of NDM- and KPC-producers to meropenem. A significant meropenem-induced increase in the expression of the outer membrane protein A was only observed in IMP-producers, thus demonstrating that carbapenemase-mediated resistance relies on far more complex mechanisms than simple inactivation of the antibiotic.

## Introduction

Resistance to antimicrobial agents has been increasing among *Enterobacteriaceae*, to the point where resistance to multiple antibiotic classes is not uncommon. One such class is the quinolones, broad-spectrum antimicrobial agents used to treat various bacterial infections, but resistance against these compounds has increased in the clinical field^1^. Carbapenem agents are broad-spectrum β-lactam antibiotics usually considered as the last option for effective treatment for infections of resistant pathogens. Meropenem, for instance, is the most widely used clinical carbapenem and is often administered as a prophylactic treatment for patients with blood stream infections by multi-drug resistant (MDR) bacteria^2^. Thus, the emergence and spread of carbapenem-resistant *Enterobacteriaceae* (CRE) has created a serious threat to public health^1^.

The primary mechanisms of carbapenem resistance in *Enterobacteriaceae* is the acquisition of carbapenemase-encoding genes e.g. *bla*_KPC_, *bla*_IMP_ , *bla*_NDM_ and *bla*_OXA-48-like_^3^. These carbapenemases are a large group of enzymes that catalyse the hydrolytic opening of the four-membered ring characteristic for β-lactam antibiotics such as carbapenems or cephalosporins and penicillins^4,5^. β-lactamases are divided into four groups based on amino acid sequence homology (Ambler Class A-D), and carbapenemases are found in Classes A, B and D^4–6^. Class A carbapenemases, such as *Klebsiella pneumoniae* carbapenemase (KPC), require a serine residue to initiate the hydrolytic reaction. KPC was first isolated in *K. pneumoniae* in the United States, and has since disseminated worldwide in several *Enterobacteriaceae*^7,8^. Class B carbapenemases, commonly referred to as metallo-β-lactamases (MBLs), rely on metal ions in their active site to activate a bound water molecule during β-lactam ring hydrolysis. In contrast to other carbapenemase classes, for which clinically useful inhibitors are available, no known MBL inhibitors have yet reached the clinical phase^9–15^. MBLs thus pose a grave danger to health care. They are subdivided, based on sequence similarity and metal ion requirement in as many as four groups, B1-B4^4,5,16^. Two of the clinically most significant MBLs that have been identified in *Enterobacteriaceae* are the imipenem-hydrolysing β-lactamase (IMP) and the recently emerged New Delhi MBL (NDM)^17^. IMP-producing *Enterobacter cloacae* is the predominant CPE in Australia^18^. Most NDM-producing pathogens carry a diversity of resistance mechanisms and are broadly resistant to β-lactams and other drug classes^19^. NDM-1 and its variants are predominantly associated with *Enterobacteriaceae*, especially in *K. pneumoniae*, *E. coli* and *E. cloacae*^20^. Resistance to the fluoroquinolone ciprofloxacin can develop via an SOS response^21^. Further, mutations within the quinolone-resistance-determining region (QRDR) have previously been associated with resistance towards quinolone-based antibiotics^22^.

Bacterial protein expression changes in response to a large variety of stress conditions, including exposure to antibiotics^23^. In the clinical setting, meropenem has been used more widely than other carbapenems^2^. Meropenem also has superior stability on comparison to other carbapenems, including imipenem^24^. Here, we challenged antibiotic-resistant bacteria by culturing them in the presence of sub-minimum inhibitory concentrations (MICs) of meropenem. Liquid chromatography coupled with tandem mass spectrometry (LC-MS/MS) has various applications regarding pathogen identification and characterisation^25^, and was here used to observe changes to the bacterial proteome when placed under antibiotic stress.

The number of molecules to be analysed, as well as their dynamic range can be very large, posing a considerable challenge for traditional proteome data analysis. Sequential Window Acquisition of All Theoretical mass spectra (SWATH) is a rapid data-independent MS/MS acquisition method that can help overcome this challenge by identifying and quantifying a wide range of fragments^26,27^. By repeatedly cycling through sequential isolation windows over the entire chromatographic elution range, the SWATH acquisition strategy is capable of generating a complete documentation of the fragment ion spectra of all detectable sample analytes where the precursor ion signals are within the defined m/z versus retention time window^27^. Recent studies have demonstrated the changes of bacterial proteomes of antibiotic-resistant bacteria^28,29^. Here, carbapenem-resistant *E. coli* strains were chosen to probe the effect of antibiotics such as meropenem and ciprofloxacin on the proteome of these organisms. We developed a SWATH-based method to perform bacterial proteome analysis to provide insight of the responses of bacterial protein expression as a consequence of antibiotic pressure.

## Results

### Optimization of the bacterial lysis method

The matches of the identified proteins from LC-MS of IMP-producing Ec1 treated with three different lysis methods were 457 proteins using mechanical disruption in 50 mM ammonium bicarbonate, 216 proteins using mechanical disruption in 8 M thio-urea, and 51 proteins using boiling and 4% SDS in 100 mM Tris-Cl and 100 mM DTT (data not shown). Since the mechanical disruption in 50 mM ammonium bicarbonate appeared to be the most effective method it was used for the following experiments.

### Minimum inhibitory concentration (MIC) and genotypic characterisation of quinolone resistance

In order to establish suitable antibiotic concentrations, the MICs of meropenem and ciprofloxacin were determined for each isolate used in the study, including the positive control (Table 1). For meropenem, the MIC values ranged from 0.38 to >32 µg/mL, while the ciprofloxacin MICs were mostly >32 µg/mL. The only mutations identified in the QRDR of each isolate are located in the *gyrA* protein and are Ser-83→Leu and Asp-87→Asn. No mutations were observed within the *gyrB, parC* and *parE* proteins.

**Table 1:** MICs of carbapenemase-producing Enterobacteriaceae.

Carbapenemase-producing *E. coli*	References	Carbapenemase variant	MIC (μg/mL)		Antibiotic concentration to grow the bacteria (μg/mL)	
			MEM	CIP	MEM	CIP
IMP-producing *E. coli*						
IMP Ec1	^47,56^	IMP-4	3	>32	2	32
IMP Ec2	^47^	IMP-4	8	>32	4	32
IMP Ec3	^47^	IMP-4	2	>32	1.5	32
NDM-producing *E. coli*						
NDM Ec1	^57^	NDM-1	32	>32	16	32
NDM Ec2	^49,58^	NDM-3	4	>32	3	32
NDM Ec3	^48^	NDM-6	32	>32	24	32
KPC-producing *E. coli*						
KPC Ec1	^59^	KPC-3	4	>32	2	32
KPC Ec2	^59^	KPC	0.38	>32	0.3	32
KPC Ec3	^59^	KPC	1.5	>21	1	32

### Quantitative analysis by SWATH Acquisition

In the following sections, the proteins of the first and second biological replicates are described in detail. A third replicate was performed later, but showed considerable variability to the earlier sets due to the instrumental differences, especially column and variation in the software used for the analysis. Thus, this set was analysed separately and presented as supplementary data.

**Figure 1:**
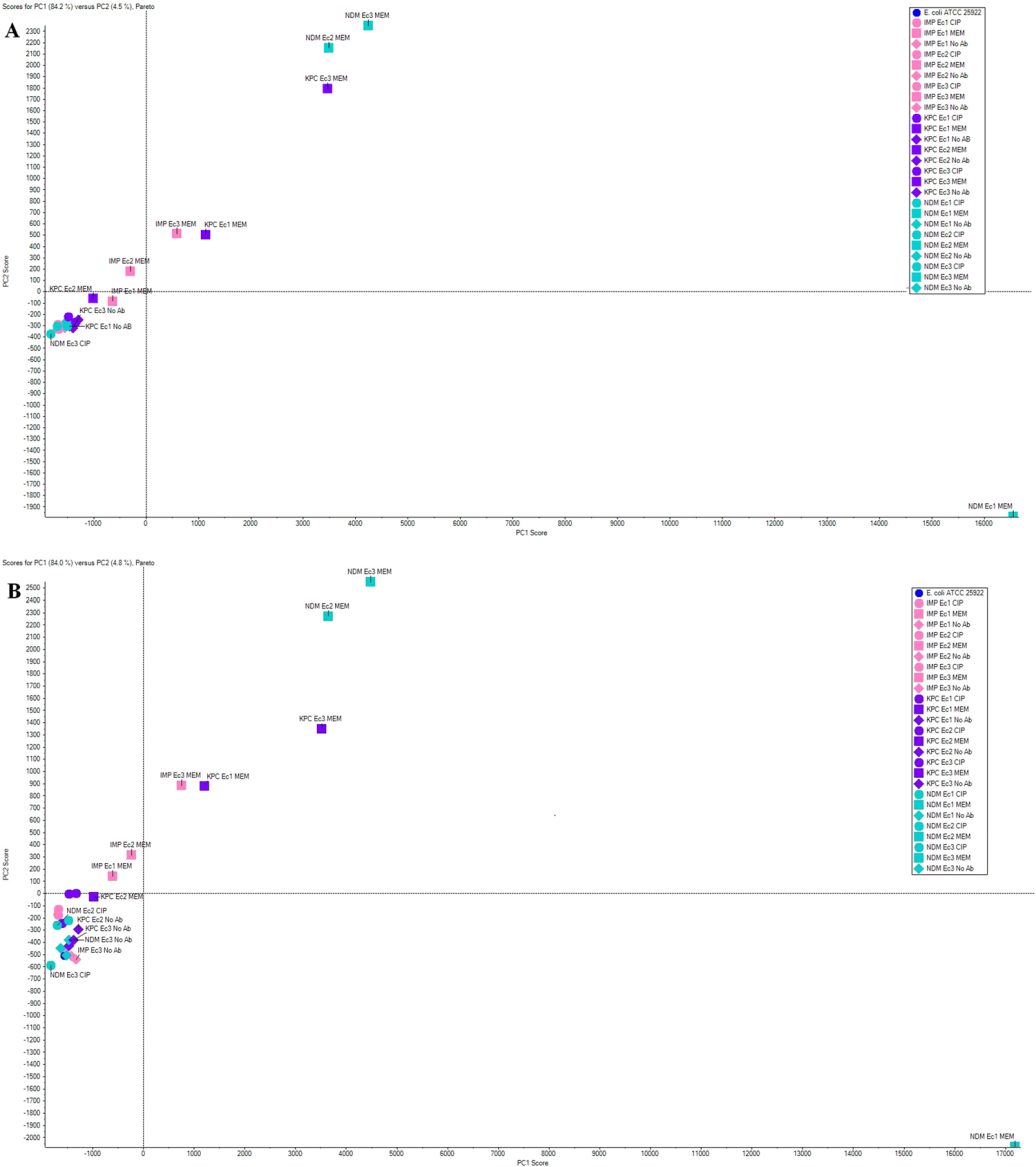
Principal Component Analysis Scores plot. A combined analysis was performed on all samples grown in different culture conditions – without antibiotic (No Ab), with meropenem (MEM) at sub-MIC or with ciprofloxacin (CIP) at sub-MIC. One control sample (*E. coli* ATCC 25922) and three strains each of IMP-, KPC- or NDM-producing *E. coli* were subjected to each culture condition. Samples were analysed via PCA, with the Scores plot representing the gross differences between samples as distances from the origin and from other samples. Results are shown for (**A**) first replicate and (**B**) second replicate.

**Figure 2:**
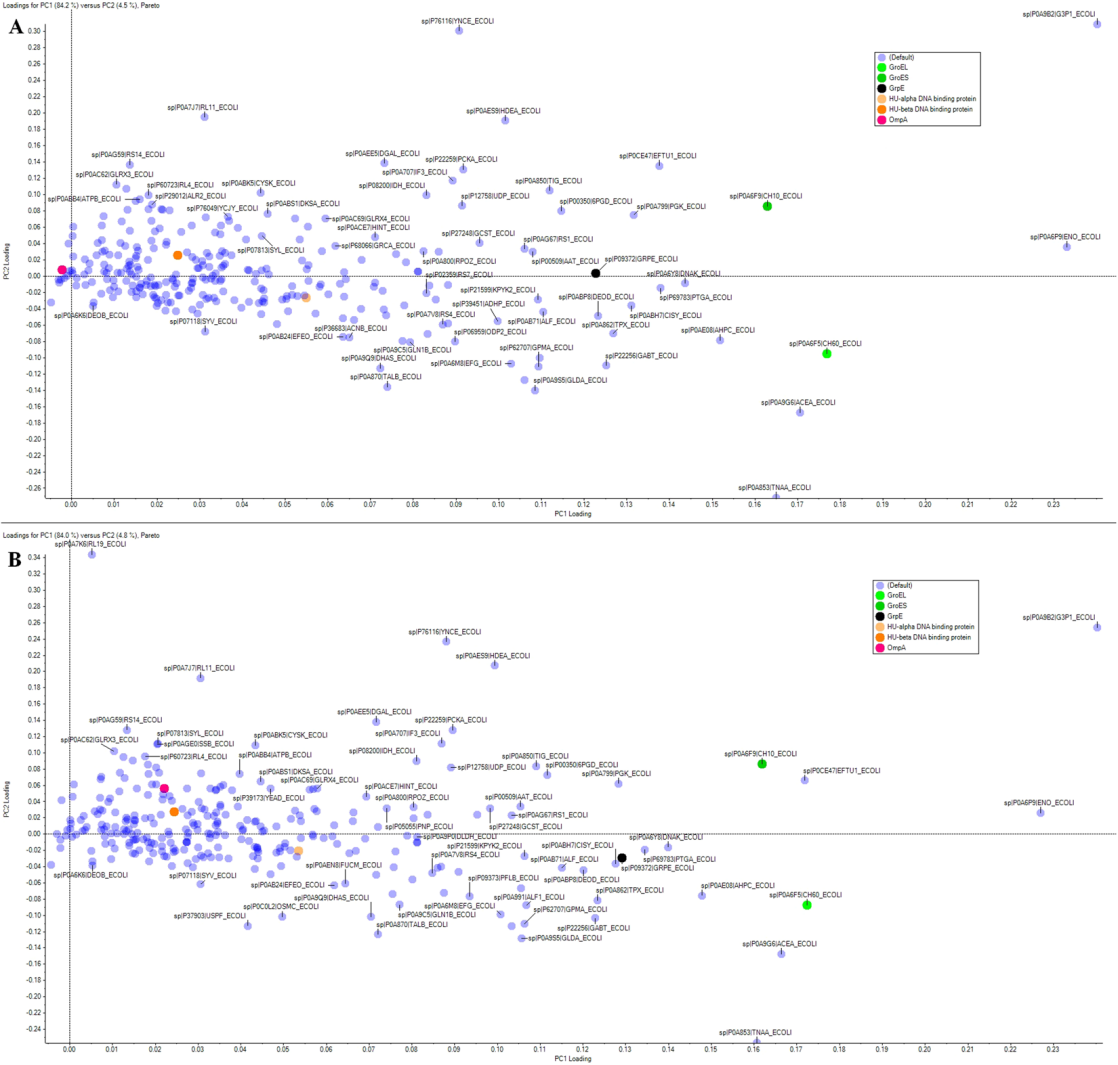
Principal Component Analysis Loadings plot for the combined analysis. Proteins with large changes in relative expression level were analysed using PCA, with the Loadings plot representing relative differences between expression levels of a given protein across samples. Proteins of interest discussed in-text are differentially coloured. Results are shown for (**A**) first replicate and (**B**) second replicate.

The Scores plot (Fig. 1) shows a summary of the relationship among samples. A Loadings plot was used to provide insight into variables that lead to sample clustering and illustrate which compounds were up- or down-regulated (Fig. 2). The expression pattern of proteins in all CPEs compared here, without antibiotic treatment, was comparable to that of the control strain, *E. coli* ATCC 25922 (Fig. 1).

The Scores plot (Fig. 1), shows that there are significant changes in the overall protein expression pattern in samples treated with meropenem. For instance, NDM-producing *E. coli* grown in the presence of ciprofloxacin or without an antibiotic was located within the same bottom-left quadrant in the plot. However, the same *E. coli* strain, grown in the presence of meropenem was located in the diagonal quadrant (top right). The PCA Loading plot for the combined analysis of all samples with the three different treatments, shown in Fig. 2, demonstrates that there are numerous proteins that underwent significant changes when treated with antibiotics, especially with meropenem. While several of these proteins uncharacterised, a few notable bacterial metabolism proteins could be identified. Their expression patterns are described in the following sections.

### Outer membrane protein A expression

**Figure 3:**
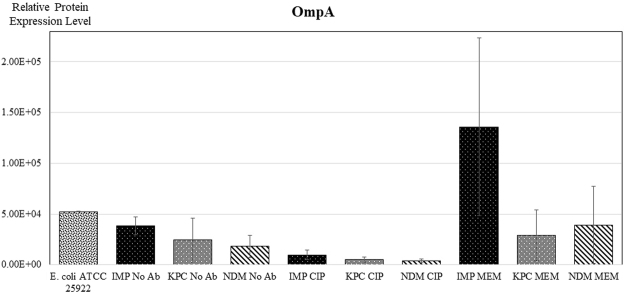
Relative comparison of expression of OmpA protein between samples. Results are from experiments performed in duplicate - three strains of each carbapenemase-type (IMP, KPC or NDM) were analysed under each condition (growing with No Ab, MEM or CIP). *E. coli* ATCC 25922 was included as a control. Results are mean ± SD.

Outer membrane protein A (OmpA) constitutes a group of proteins in the outer membranes of Gram-negative bacteria that include heat-modifiable and surface-exposed porin proteins^30^. As illustrated in Fig. 3, the expression of OmpA is substantially higher in IMP-producers when treated with meropenem, while there was no significant change observed when treated with ciprofloxacin. Interestingly, the Class A β-lactamase KPC and the Class B MBL NDM appear to display a similar effect on OmpA expression; in both cases the presence of antibiotics, especially ciprofloxacin, leads to some decrease in OmpA production.

### HU DNA-binding protein expression

**Figure 4:**
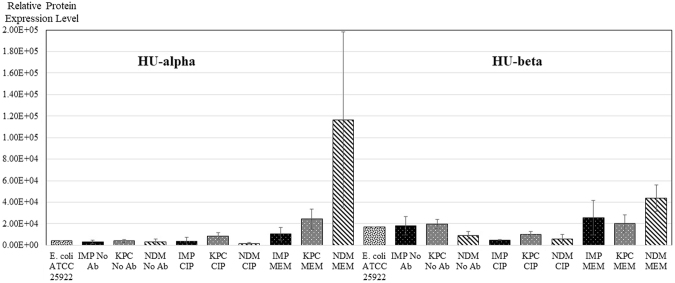
Relative comparison of expression of HU-alpha and beta DNA binding proteins between samples. Results are from experiments performed in duplicate - three strains of each carbapenemase-type (IMP, KPC or NDM) were analysed under each condition (growing with No Ab, MEM or CIP). *E. coli* ATCC 25922 was included as a control. Results are mean ± SD.

HU DNA-binding proteins are capable of wrapping DNA to stabilize it and thus prevent its denaturation under extreme environmental conditions. These histone-like DNA-binding proteins are made up of an alpha and a beta chain^31,32^. The expression pattern of both chains was affected similarly when meropenem was added, inducing by far the most dramatic effect, in NDM-producers (Fig. 4). Interestingly, and in stark contrast to the effect on OmpA, NDM-producing, and to some extent also KPC-producing strains have greatly enhanced expression of HU DNA-binding proteins, whereas IMP-producing strains are only marginally affected.

### Expression of GroEL/GroES and GrpE

**Figure 5:**
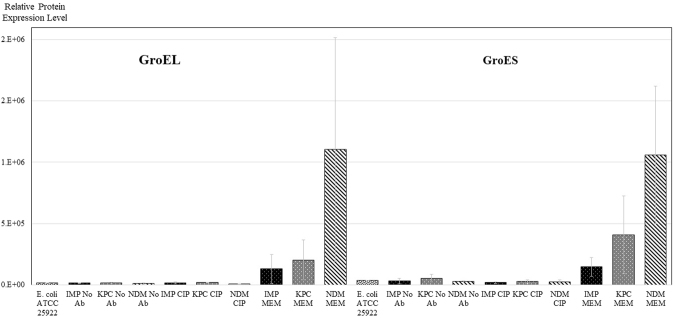
Relative comparison of expression of chaperonin protein complex GroEL/GroES between samples. Results are from experiments performed in duplicate - three strains of each carbapenemase-type (IMP, KPC or NDM) were analysed under each condition (growing with No Ab, MEM or CIP). *E. coli* ATCC 25922 was included as a control. Results are mean ± SD.

GroEL/GroES is a protein complex that belongs to the chaperonin family of molecular chaperones and interacts with 250 proteins in *E. coli*^33^. The expression of GroEL/GroES mirrors largely that observed for the HU DNA protein with meropenem triggering a significant overexpression in particular in NDM-producing CPEs (Fig. 5). There was no apparent difference in GroEL/GroES expression in samples treated with ciprofloxacin and without that antibiotic.

**Figure 6:**
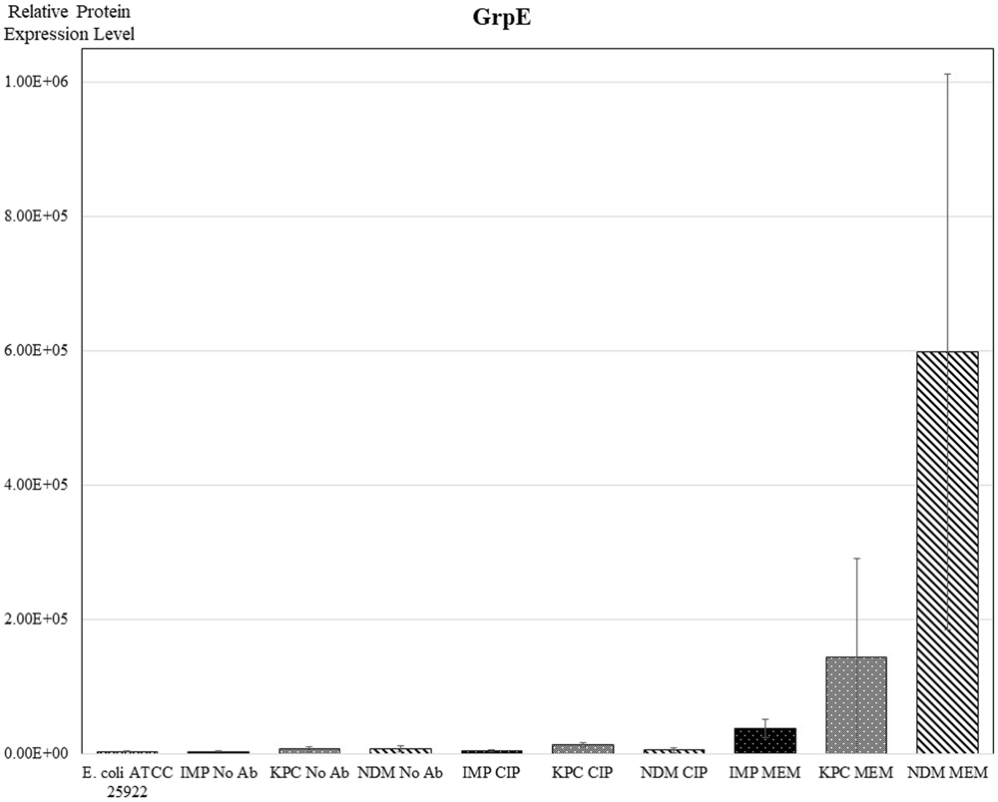
Relative comparison of expression of GrpE protein between samples. Results are from experiments performed in duplicate - three strains of each carbapenemase-type (IMP, KPC or NDM) were analysed under each condition (growing with No Ab, MEM or CIP). *E. coli* ATCC 25922 was included as a control. Results are mean ± SD.

The protein GrpE participates actively in the response to hyperosmotic pressure and heat shock by preventing the aggregation of stress-denatured proteins^34^. Again a similar effect on its expression is observed with only meropenem initiating significant overexpression, in particular for the NDM-producing strain (Fig. 6).

## Discussion

It is known that exposure to antibiotics may trigger an array of responses in pathogens in their attempt to lessen or avoid the detrimental effects of these agents. As an example, quinolones are powerful broad-spectrum antimicrobials used for the treatment of a wide variety of community-acquired and nosocomial infections^35^. The most common mechanism of resistance to quinolones in *E. coli* includes alterations in genes located in the QRDR and that encode subunits of the quinolone targets, *i.e*. DNA gyrase (*gyrA* and *gyrB* genes) and topoisomerase IV (genes *parC* and *parE*)^22^. In this study, all CPE isolates are resistant to the quinolone ciprofloxacin with a MIC of >32 µg/mL (Table 1). The two amino acid substitutions present in the *gyrA* protein (Ser-83→Leu and Asp-87→Asn) are commonly reported for quinolone-resistant pathogens^22^.

Antibiotics may also affect the expression levels of distinct proteins. This was illustrated here, as CPE protein expression patterns without antibiotic pressure were comparable to the control (Fig. 1), while antibiotic exposure lead to significant proteome differences between CPE and the antibiotic-susceptible control. A similar approach has been done by Suh and colleagues, using ESBL-producing *Klebsiella pneumoniae* with antibiotic pressure (ampicillin, streptomycin and doxycycline)^28^. They found that antibiotic pressure induced expression of a set of proteins, most of which were outer membrane proteins.

As an example from a previous study, exposure to the antibiotic tetracycline lead to an up-regulation of the outer membrane protein OmpA^36^. Outer membrane proteins play an important role in the membrane permeability and efflux systems of bacteria, affecting drug permeation across the bacterial membrane significantly^37^. Outer membrane permeability is regulated by porin proteins and it has been shown that the expression levels of porins may be regulated by the concentration of antibiotics in the environment, thereby contributing to antibiotic resistance^36^. OmpA is one of the major outer membrane proteins that is involved in the physiological adaptation in antibiotic exposure^36^. Here, we observed that the presence of ciprofloxacin reduced OmpA expression across all carbapenemase-producing *E coli* (Fig. 3). This may indicate that ciprofloxacin may be effective in reducing biofilm formation, which is often caused by the overexpression of OmpA in *E. coli*^38^. In comparison, the effects of meropenem exposure on OmpA expression varied depending on the carbapenemase involved. Meropenem exposure lead to an increase in OmpA expression for IMP-producing *E. coli* only. As meropenem targets and inhibits bacterial cell wall synthesis, the increase in OmpA expression may highlight a bacterial strategy to adapt to stress invoked by that antibiotic. This observation suggests that OmpA may be a target to combat β-lactam resistance at least among IMP-producing CPEs - considering the similar catalytic efficiency of IMP and NDM towards carbapenems, it is unknown why OmpA expression is considerably more pronounced in IMP-producing strains.

The differences in the proteome of IMP-producing CPEs on the one hand, and NDM- and KPC-producing ones on the other are also observed when the levels of the small, thermostable HU DNA protein are investigated (Fig. 4). In *Enterobacteriaceae*, including *E. coli*, HU is associated with the bacterial nucleoid and is a heterotypic dimer (HUαβ) that is composed of two closely related subunits encoded by the *hupA* and *hupB* genes^31^. HU plays an important role in maintaining the negative super-coiling density in the bacterial cell^39^. Ciprofloxacin had very little effect on the expression levels of the HU protein in all strains tested, while meropenem lead to increases in expression levels for NDM- and KPC-producing strains, particularly for the α subunit.

The effects observed for the HU proteins are similar to those recorded for the GroEL/GroES chaperonin complex (Fig. 5), indicating that there may be a link between the HU DNA-binding protein and chaperonins for bacterial survival. Again, while the addition of ciprofloxacin triggered no effect in any of the analysed strains, the presence of meropenem caused a drastic up-regulation of the chaperonins for all strains, particularly for some of the NDM-producing CPE. Chaperones are essential as they are required for the folding, disaggregation, transport and function of proteins^40^. They help to buffer the effects of environmental changes, as observed by a marked increase in expression levels under various stress conditions such as antibiotic pressure^41^. Bacteria facing high mutational loads were shown to be fitter when overexpressing GroEL/GroES chaperonins^42^. GroEL/GroES are chromosomally encoded in *E. coli*. Furthermore, NDM-producers generally have GroEL/GroES genes within the Tn125 on the NDM plasmids^43,44^. While these plasmid-borne GroEL/GroES chaperonins may have contributed to the enhanced expression of these proteins in the NDM-producers, their high levels also pinpoint a major reason why NDM-producing CPEs have a higher survival chance than KPC- or IMP-producers when exposed to meropenem.

Finally, the trends observed for OmpA, the HU DNA binding protein and the chaperonins are also mirrored in the behaviour of the heat shock protein GrpE (Fig. 6). Together with DnaK and DnaJ, GrpE forms a cellular chaperone machinery which functions to repair heat-induced protein damage and likely plays a key role in a homeostatic mechanism that controls the expression of heat shock genes in response to environmental stress^45^. It has been shown that the overexpression of DnaK/DnaJ/GrpE helped in promoting cell survival after aminoglycoside exposure. It was also suggested that the membrane potential can be partly rescued by an increased cellular chaperonin protein folding capacity since membrane potential is affected during aminoglycoside-induced membrane disruption^46^.

In summary, here we used a method for the qualitative and quantitative analysis of bacterial proteins involved in antibiotic resistance using LC-MS/MS and SWATH. This approach has provided comprehensive information about the proteome of antibiotic resistant bacteria, which helped in understanding bacterial adaption and the role of antibiotic pressure in antibiotic resistance. The most important finding was the differential effect of meropenem on the proteome of CPEs producing different β-lactamases. Specifically, the magnitude in the increase of proteins relevant to bacterial cell adaptation and survival under antibiotic pressure was the greatest in NDM producers, followed by KPC and IMP producers. NDM producers thus were most likely to have better ability to adapt under such antibiotic pressure than KPC and IMP producers. The mechanism underlying the different effects of different carbapenemases on the proteome of CPEs is currently obscure and is in contrast to the relatively conserved *in vitro* enzymatic mechanism of IMP and NDM, which differs from that of KPC (*i.e*. the former enzymes are MBLs, whereas KPC is a metal-independent MBL). Hence, there seems to be no simple correlation between isolated enzymatic properties and the cellular response to antibiotic pressure. The observation that NDM triggers a drastic up-regulation in a number of essential proteins exacerbates this enzyme’s threat to health care as it is not only a very efficient β-lactamase with a very broad substrate range, but it is also capable of triggering a diverse response to antibiotic stress that enhance the viability of the pathogen considerably. It is important to note that the experiments performed here allowed only for a comparative (rather than absolute) quantitative analysis. This allowed for us to compare relative protein expression levels between samples within each experiment, but does not give an absolute measure of the protein. The limitation of using this as an experimental design is demonstrated by the large error bars observed here. When comparing results of the two replicates separately, there was little variation; however, when the two replicates’ results were combined, larger variation was seen as the relative comparisons are not the same across the replicates. Future refining of this method by spiking in a known amount of a certain protein (e.g. Bovine Serum Albumin) is expected to allow for absolute protein quantitation, giving a more appropriate way to compare replicate results.

Nonetheless, this method has provided an excellent baseline on which to build future experiments on. Methods like SWATH have been very useful in identifying the differential effect of various β-lactamases on the proteomes of a particular pathogen (*i.e. E. coli*), and may also play an integral part in identifying factors that contribute towards the enzyme-specific response of the proteome when subjected to antibiotic stress. Other further studies, such as multiple reaction monitoring (MRM) will be necessary to map the role(s) of each of the specific key proteins identified here in enhancing antibiotic resistance. Efforts towards this goal are currently in progress and may pave the way for novel strategies to combat antibiotic resistance.

## Materials and Methods

### Bacterial strains, minimum inhibitory concentration (MIC) and culture condition

A total of nine genotypically characterized carbapenemase-producing *E. coli* isolates were used in this study (Table 1). The isolates were comprised of three strains each of IMP-4-producing *E. coli*^47^, NDM-producing *E. coli*^48–50^ and KPC-producing *E. coli*^51^. In addition to these isolates, purified IMP-1 obtained from a plasmid construct (*bla*_IMP−1_ construct in BL21 *E. coli*) was used as a positive control during the optimisation of the methods. E-tests (bioMerieux) was used to determine the meropenem and ciprofloxacin minimum inhibitory concentrations (MICs) of the study isolates on Mueller-Hinton agar, and results were interpreted in terms of EUCAST breakpoints^52^. The nine study isolates were analysed under three different culture conditions, *i.e*. (i) in the absence of antibiotics, (ii) in the presence of ciprofloxacin and (iii) in the presence of meropenem, both at concentrations below their respective MICs or sub-MICs (Table 1). The isolates were cultured on Luria Bertani agar overnight. Subsequently, a single colony from each isolate was cultured in Mueller-Hinton broth. An optimization step to establish optimum bacterial growth condition to obtain the highest number of identifiable proteins was also performed. The number of identified proteins from an overnight bacterial culture and from bacteria harvested at 0.5 of OD_600_ were compared. In the overnight culture, outer membrane proteins were abundant, reducing the number of other identified proteins. A bacterial culture harvested at OD_600_ ~ 0.5 thus provided a better sample for protein analysis (data not shown). Therefore, the culture of *E. coli* in Mueller-Hinton broth without or with appropriate antibiotics for 4 hours with shaking at 250 rpm at 37 °C to reach the exponential growth phase at OD_600_ of 0.5 was used.

A preliminary step was performed to determine the sub-MICs antibiotic concentration in Mueller-Hinton broth by growing the bacteria at various antibiotic concentrations. Meropenem MICs of study isolates were variable, ranging from 0.38 to 32 µg/mL. In contrast, all the study isolates had ciprofloxacin MICs >32 µg/mL. The optimum meropenem and ciprofloxacin sub-MICs were defined where the study isolates were capable to grow robustly at highest sub-MICs. The empirical formula for meropenem sub-MIC was MIC × 0.75. Where meropenem sub-MICs were calculated to three decimals, the sub-MICS were rounded to the closest measurable sub-MICs (Table 1). The maximum scale of ciprofloxacin MIC with the E-test was 32 µg/mL; therefore, the ciprofloxacin concentration used to grow the study isolates was 32 µg/mL. Once the method was optimised, biological duplicates were performed to each sample to understand the reproducibility of the methods and results. Further a second set of biological duplicates were performed to the study isolates in the presence of meropenem only to confirm the variability of bacterial protein expression response under meropenem pressure.

### Genotypic characterisation of quinolone resistance

QRDR mutations were detected by PCR and sequencing of the *gyrA*, *gyrB*, *parC* and *parE* genes^53^. The PCR products were sequenced using the BigDye Terminator v3.1 cycle sequencing kit in the Applied Biosystems 3730XL sequencer. The reference sequences used were from wild-type *E. coli gyrA* (Genbank accession no. X06373), *gyrB* (GenBank accession no. X04341), *parC* (GenBank accession no. M58408) and *parE* (GenBank accession no. AE000385).

### Preliminary work to obtain the most number of detected proteins

In order to obtain the highest number of detected proteins from LC-MS/MS, three different bacterial lysis methods in a single isolate, IMP-4-producing *E. coli* (Ec1), were compared. They were (i) mechanical disruption using TissueLyser (TissueLyser II, QIAGEN) in 50 mM ammonium bicarbonate lysis buffer, (ii) lysis in 8 M thiourea buffer, and (iii) boiling the bacterial suspension in 4% SDS (Bio-Rad) in 100 mM Tris-Cl and 100 mM DTT at 110 °C for 10 minutes. Following lysis, the samples were centrifuged for 10 min and the supernatant was transferred to protein low-binding microtubes. The proteins were quantitated and trypsin digested, desalted and subsequently analysed in the LC-MS/MS as described below. The LC-MS/MS data were then processed on Mascot to search for protein matches (www.matrixscience.com). The lysis method which yielded the highest number of protein matches was selected for the experiment.

### Protein quantification using bicinchoninic acid assay (BCA)

Proteins were quantified in triplicates using the BCA assay (Sigma-Aldrich) and bovine serum albumin (BSA) as standard. After incubation at 37 °C for 30 min, a microplate reader (BMG Labtech, Ortenberg/Germany) was used to determine the protein concentration at 562 nm.

### Reduction and alkylation

Aliquots of 1 μg protein/μL were mixed with ammonium bicarbonate (100 μl of 50 mM) for 10 s. Ten microliters of 20 mM DTT/bicarbonate were added to each sample and incubated for 1 h at 60 °C. Iodoacetamide (10 μl of 1 M in 100 mM bicarbonate) was added to each tube and incubated for another hour at 37 °C, protected from light.

### Trypsin digestion and desalting

Samples were digested with Trypsin Gold (Promega, USA) to cleave proteins into peptides on the carboxyl side of amino acid residues lysine and arginine. Following digestion, 0.1% formic acid (100 μL) was added and samples were centrifuged (15,000 rpm for 15 min) using a 10 kDa size exclusion membrane (PALL, Nanosep Cheltenham Vic, Australia). The flow-through was retained for a desalting step using a 3 mm piece of an Empore C18 (Octadecyl) SPE Extraction Disk. The disk was excised and placed in a gel loader tip and 5 μL of a POROS R3 slurry were added to form a micro-column. This column was washed with trifluoroacetic acid (20 μL, 0.1% in water). Peptides were eluted from the micro-column by three washes of acetonitrile (20 μL, 0.1% formic acid). Elutes were pooled and samples were dried at room temperature in a vacuum evaporator for 45 min. Subsequently, the samples were reconstituted with 100 µl of 0.1% formic acid in H_2_O and centrifuged for 2 min at 10,000 g to remove particulates.

### Sample analysis by LC-MS/MS

LC-MS/MS analysis of digested *E. coli* lysates was performed on a Tandem Quadrupole Time-of-Flight mass spectrometer Sciex TripleTOF 5600 (Sciex) coupled to an Eksigent 1D+ Nano LC system and a nanoFlex cHiPLC system (Eksigent) with a Nanospray III Ion Source (Sciex). Peptides were separated using a linear gradient (60 min for 5 to 80% B at 500 nL/min) of 0.1% formic acid in water and 0.1% formic acid in acetonitrile, and were delivered by a nanospray III electrospray interface (105 mm stainless steel emitter, Thermo Fisher THIES528).

Data acquisition of peptide separation using LC-MS/MS was performed using two different methods: the information-dependent acquisition (IDA) method and the data-independent (SWATH) acquisition method. Technical duplicates were performed for the IDA method.

Following the LC-MS/MS analysis, the mass spectral data generated from the IDA method were processed through two different search algorithms, Mascot (Matrix Science, v. 2.4.0) and Paragon (ABSciex, ProteinPilot Software v. 4.5.0.0, 1654). To generate an ion library, LC-MS/MS mass spectral data were firstly analysed using Mascot and the *Eubacteria* database from SwissProt for a qualitative analysis to identify and detect the presence or absence of the proteins of interest. Secondly, the analysis using Paragon involved a search against a suitable FASTA-formatted *E. coli* protein database from UniProt for the identification of peptides from the mass spectral data^54^. The data from Paragon were loaded onto PeakView (Sciex, v.1.2.0.3) to interrogate the SWATH data bank using the ion library generated in ProteinPilot. PeakView performs targeted and non-targeted data processing and generates extracted ion chromatograms (XIC). The data were then transferred to Markerview (Sciex, v. 1.2.1.1) for result interpretation and quantitative analysis. Markerview allows for a rapid review of the data to determine up and down-regulation of protein expression^26^. The data were processed using principal component analysis (PCA), which is an unbiased multivariate statistical analysis method that compares data across multiple samples, revealing groupings among data sets and graphically present the groupings in a Scores plot.

### SWATH analysis

Specific to analyse the data from the SWATH method, three different software programs were used. Firstly, Paragon was used to streamline protein identification and quantitation by identifying hundreds of peptide modifications and non-tryptic cleavages simultaneously to build an ion library. Then, using PeakView (Sciex, v. 1.2.0.3), the SWATH data were interrogated against the ion library. PeakView allows mass spectral data to be explored and interpreted for processing accurate mass data, structural interpretation and batch analysis. MarkerView was then used to review the data to determine the up or down regulation of protein expression in the bacterial samples through the use of Principle Components Analysis (PCA), a statistical tool that produces a visual representation of patterns in a dataset. The mass spectrometry proteomics data have been deposited to the ProteomeXchange Consortium via the PRIDE^55^ partner repository with the dataset identifier PXD008019.

## Electronic supplementary material

Supplementary data
