# Supplementary material for: The use of SWATH to analyse the dynamic changes of bacterial proteome of carbapanemase-producing Escherichia coli under antibiotic pressure
Source: Sci Rep. 2018 Mar 1;8:3871. doi: 10.1038/s41598-018-21984-9 (PMC5832786; doi:10.1038/s41598-018-21984-9)
Supplement: Supplementary file 1 — Supplementary data [file 41598_2018_21984_MOESM1_ESM.pdf]

Supplementary data of the third biological duplicate of bacterial proteome data of carbapenemase-producing *Escherichia coli* and *E. coli* ATCC 25922 with meropenem pressure

Manuscript title:

The use of SWATH to analyse the dynamic changes of bacterial proteome of carbapanemase-producing *Escherichia coli* under antibiotic pressure

Hanna E. Sidjabat[a,*] Jolene Gien[a], David Kvaskoff[a], Keith Ashman[b], Kanchan Vaswani[a], Sarah Reed[a], Ross P. McGeary[c], David L. Paterson[a], Amanda Bordin[a], Gerhard Schenk[c,*]

[a] Centre for Clinical Research, Faculty of Medicine, The University of Queensland, Royal Brisbane and Women's Hospital Complex, Herston, 4029, Australia

[b] Sciex, Mulgrave, Victoria, Australia

[c] The University of Queensland, Australia, School of Chemistry and Molecular Biosciences, Brisbane Qld 4072, Australia.

Keywords: Antibiotic resistance, proteomics, SWATH analysis, carbapenemases, IMP, NDM, KPC.

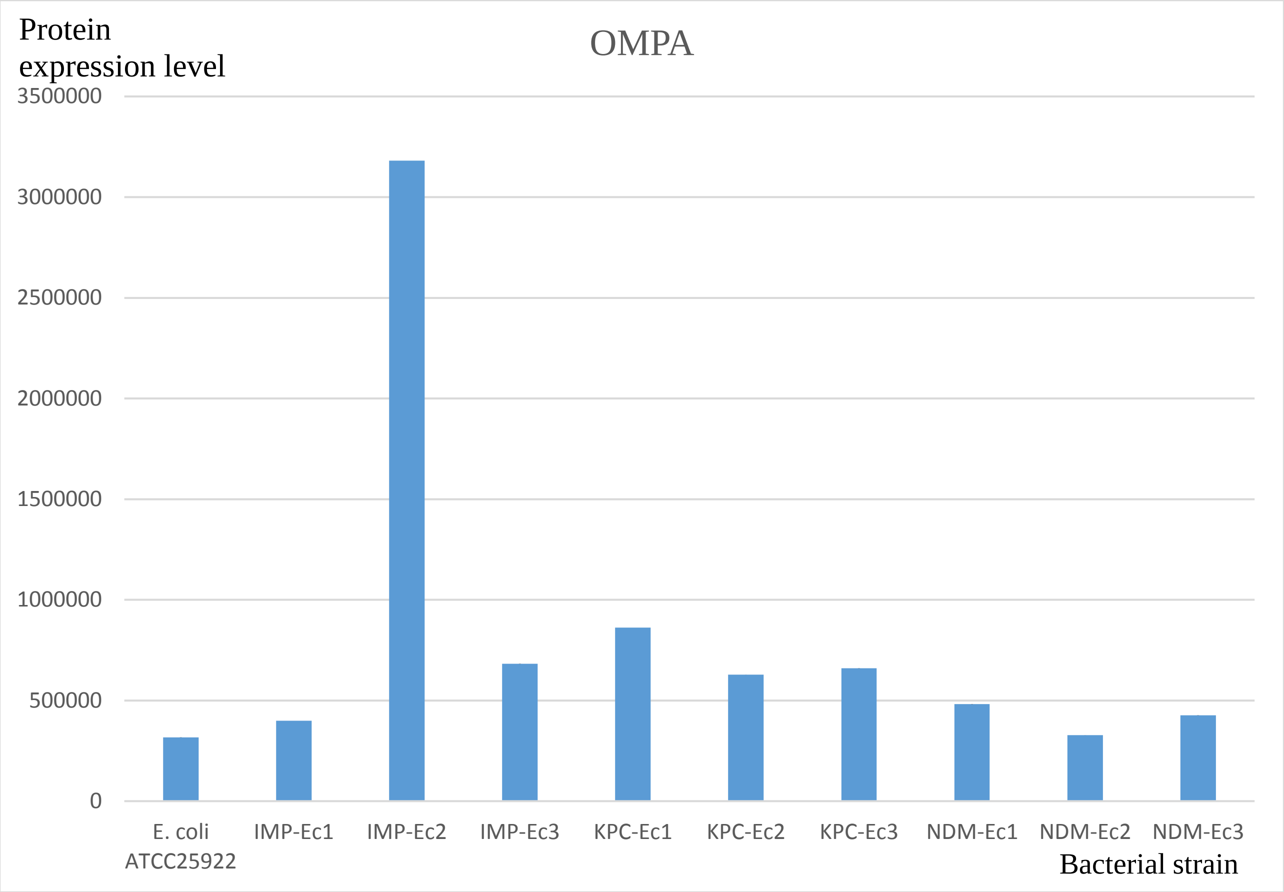

Figure S1. Expression of OmpA

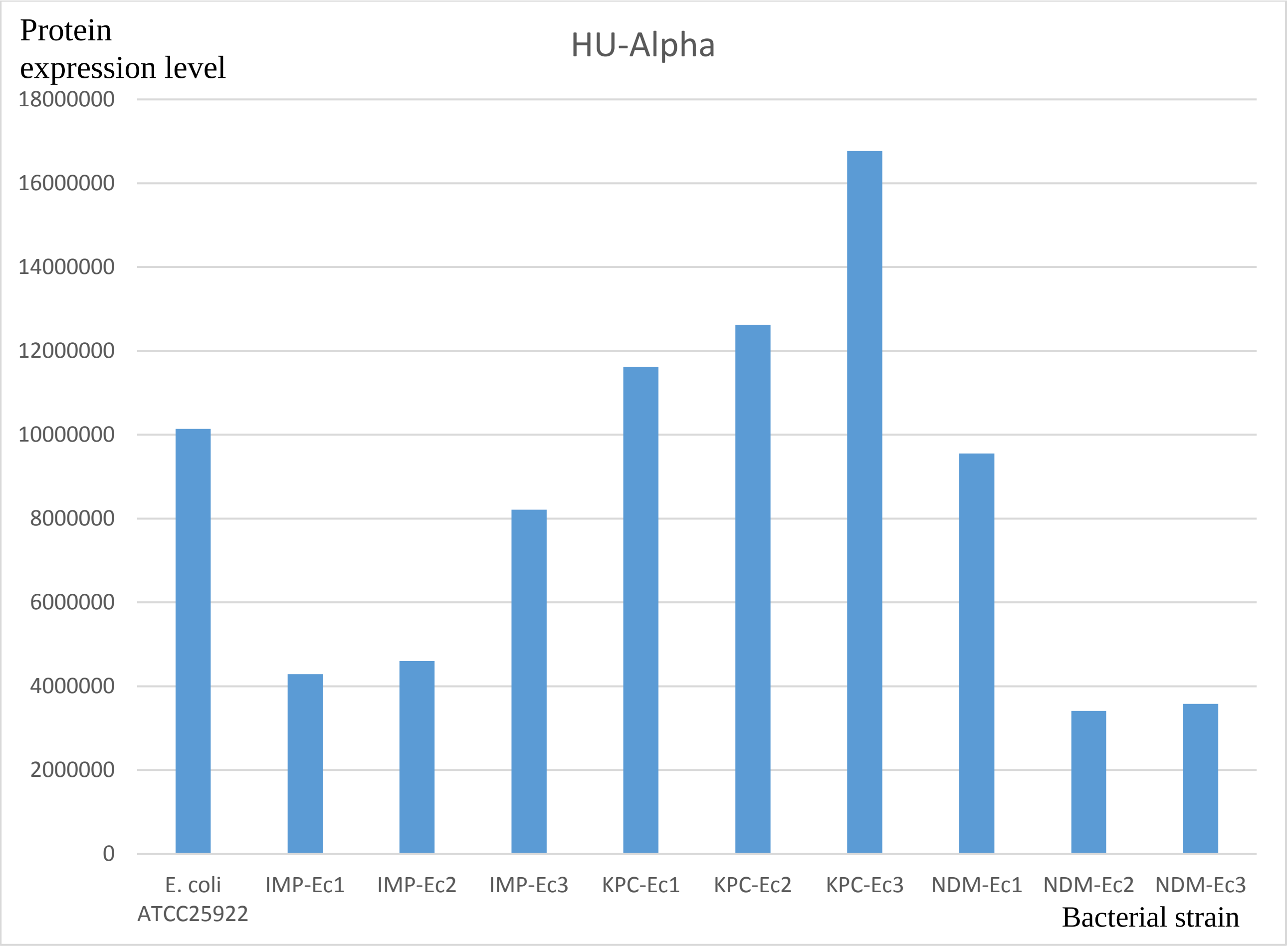

Figure S2. Expression of HU-alpha DNA binding proteins.

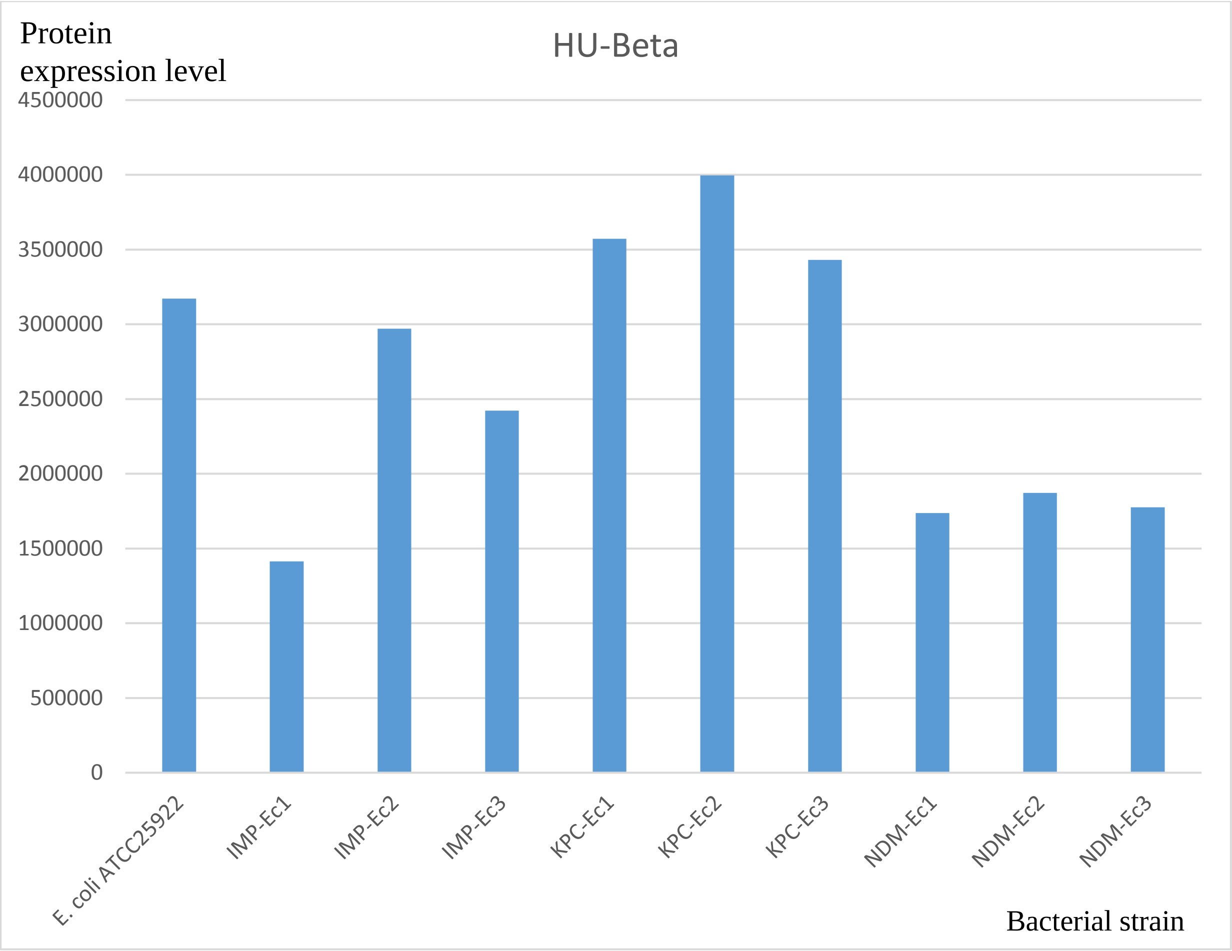

Figure S3. Expression of HU-beta DNA binding proteins.

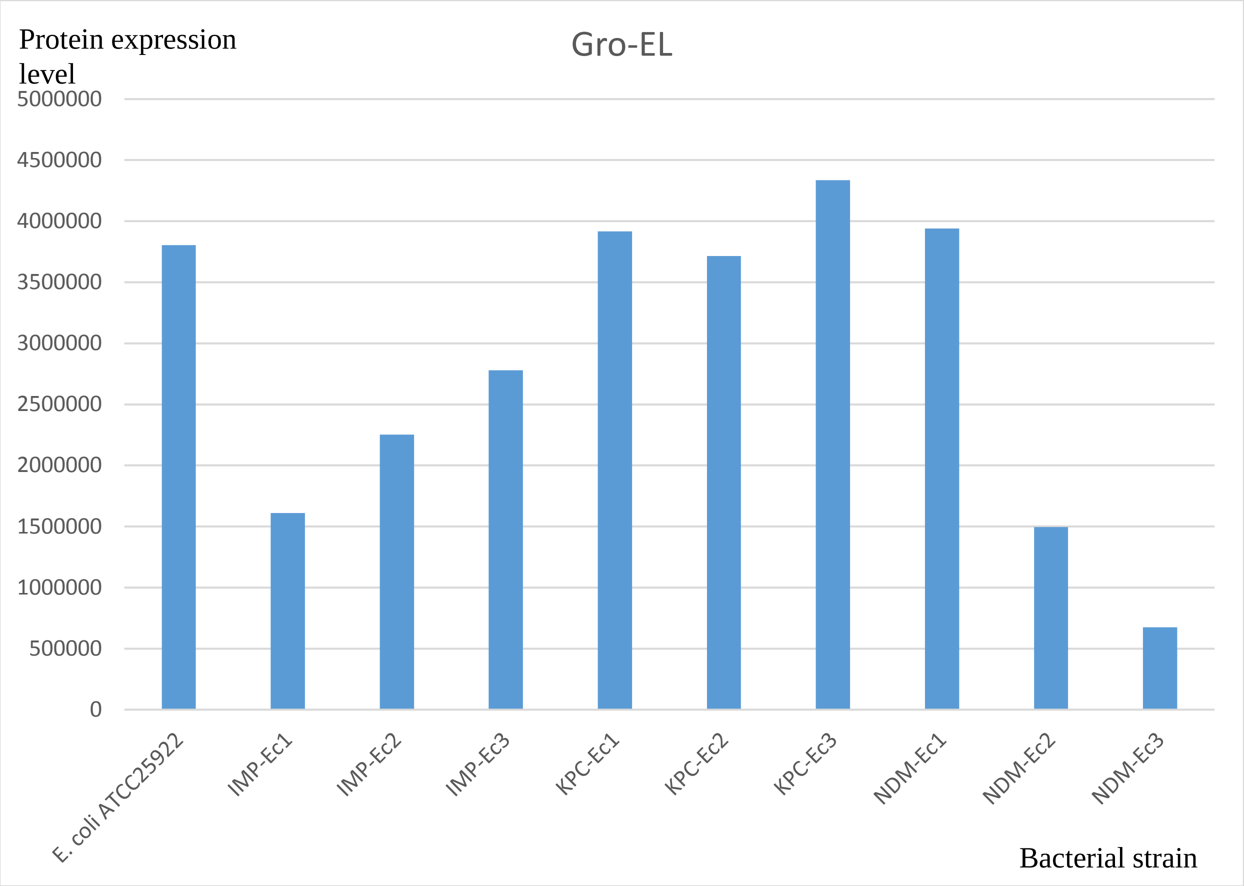

Figure S4. Expression of GroEL.

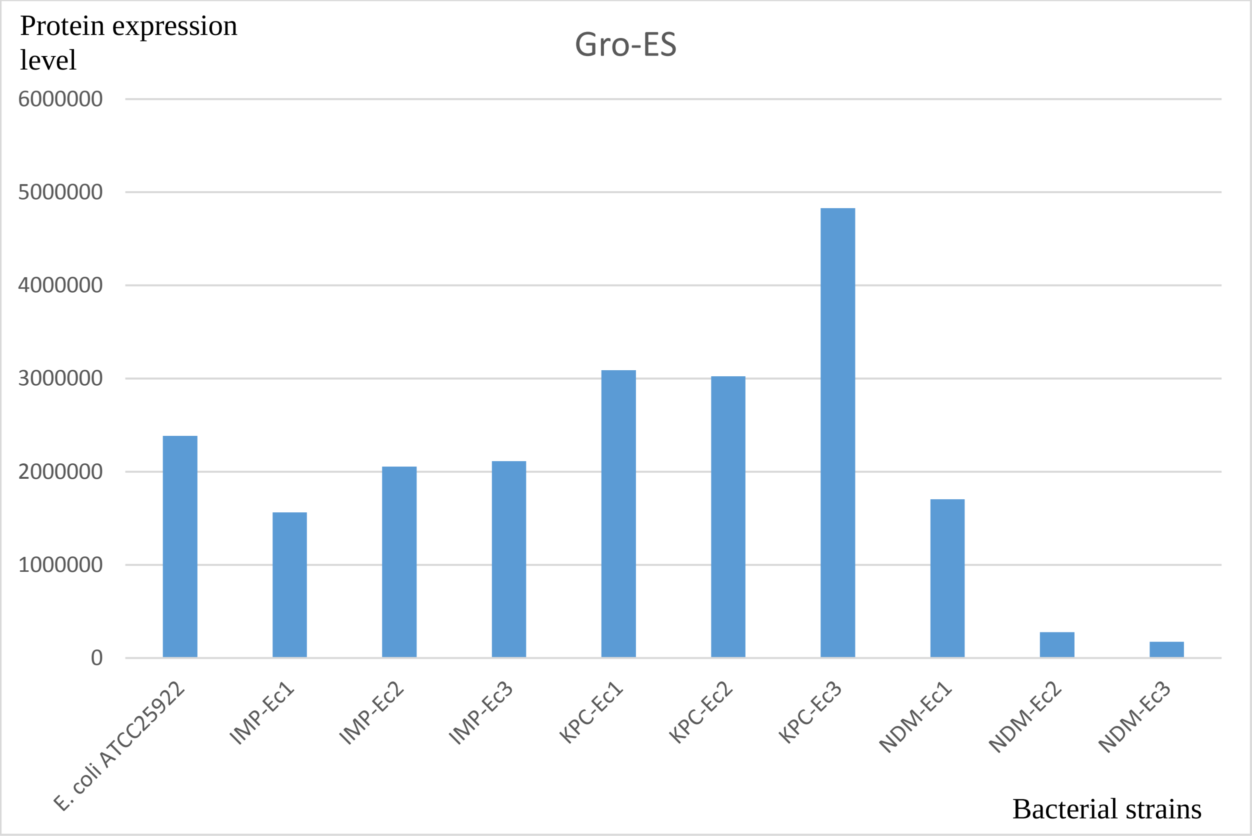

Figure S5. Expression of GroES.
